# Beyond the Liver: Brain Rap1 Signaling in Metformin’s Antidiabetic Action

**DOI:** 10.3390/biology15201810

**Published:** 2026-10-10

**Authors:** Masood Alam Khan, Mohammad Hamza Khan

**Affiliations:** 1 Department of Basic Health Sciences, College of Applied Medical Sciences, Qassim University, Buraydah 51452, Saudi Arabia; 2 Jawaharlal Nehru Medical College and Hospital (JNMCH), Aligarh 202002, India

**Keywords:** diabetes mellitus, brain, Rap1 signaling, metformin, mTOR, neural networks

## Abstract

Metformin remains a promising therapeutic drug for type 2 diabetes mellitus (T2DM) due to its glucose-lowering effects through hepatic and other peripheral mechanisms. Recent evidence shows that the brain may represent an additional site of action. The small GTPase Ras-related protein 1 (Rap1), potentially connects central metformin exposure to systemic glucose homeostasis. The current review discusses neuronal Rap1 regulation and a role in metformin’s antidiabetic effects. Preclinical findings indicate that excessive neuronal Rap1 activity may contribute to metabolic dysfunction, whereas its inhibition is related to improved glucose regulation. Recent experimental evidence further suggests that low-dose metformin reduces Rap1 activity in the ventromedial hypothalamus (VMH). Moreover, metformin also alters the excitability of steroidogenic factor-1-positive neurons that contributes to its hypoglycemic effect. Initial findings also suggest a functional relationship between Rap1 and AMPK, although the molecular mechanisms showing the mTORC1 connection remain to be clarified. This review also considers how central Rap1 signaling might interact with gut–brain–liver pathways that can affect peripheral metabolism. Human central nervous system (CNS) studies and primary RAP1A pharmacogenetic results offer indirect translational support, but direct evidence of hypothalamic Rap1 involvement in patients is currently not documented. Extending beyond previous reviews of the preclinical VMH–Rap1 mechanism, this review integrates dose–CNS exposure relationships with emerging human evidence, particularly CSF pharmacokinetics and RAP1A pharmacogenetics. These findings provide preliminary translational links, although direct hypothalamic Rap1 engagement in humans remains unproven. Future studies should elucidate circuit-specific mechanisms, determine reliable biomarkers, and assess selective pathway modulation. A better understanding of this relationship may help determine whether brain Rap1 could ultimately serve as a therapeutic target to improve metformin responsiveness in T2DM.

## Simple Summary

Metformin is the most common drug used to regulate blood glucose in people with type 2 diabetes. Although its actions are well known in the liver and other peripheral tissues, emerging evidence indicates that the brain may also play a role in its glucose-lowering effects. This review focuses on Rap1, a signaling protein involved in the regulation of metabolism within the brain. Experimental studies indicate that Rap1 action in the ventromedial hypothalamus can impact blood glucose and insulin sensitivity. Low-dose metformin, particularly, may reduce Rap1 activity and alter the function of specific hypothalamic neurons, resulting in better glucose control. However, main evidence for this mechanism presently comes from animal studies, and its importance in humans remains uncertain. The review also discusses how metformin reaches the brain, possible connections between Rap1 and other metabolic pathways, and current human evidence. Further research is essential to determine whether brain Rap1 can explain the differences in individual responses to metformin.

## 1. Introduction

Type 2 diabetes mellitus (T2DM) is a chronic metabolic disorder characterized by insulin resistance, impaired insulin secretion, and persistent hyperglycemia [1]. Although its pathophysiology is strongly associated with pancreatic β-cell dysfunction and altered metabolism in the liver, skeletal muscle, and adipose tissue [2], increasing evidence indicates that the central nervous system (CNS) also contributes to glucose homeostasis [3]. The brain responds to circulating nutrients and metabolic hormones and coordinates neural signals that influence peripheral glucose metabolism [4]. Consistent with this broader view, T2DM is frequently associated with alterations in brain structure and function [5]. Chronic hyperglycemia, oxidative stress, mitochondrial dysfunction, and blood–brain barrier abnormalities may contribute to these neurological changes [6]. The relationship is likely bidirectional, as impaired central metabolic regulation may further influence systemic glucose control [7]. Accordingly, the CNS is increasingly recognized as an important component of the integrated regulation of glucose metabolism [8]. Within the CNS, the hypothalamus has a central role in integrating nutrient and hormonal information with whole-body metabolic demands [9]. Hypothalamic neuronal circuits respond to glucose, insulin, leptin, and other metabolic signals and can influence energy balance, insulin sensitivity, and hepatic glucose production [10]. Disruption of these sensing mechanisms during obesity and T2DM may therefore contribute to impaired communication between the brain and peripheral metabolic organs. Specialized hypothalamic glial cells such as tanycytes also contribute to nutrient and glucose sensing and may participate in metabolic communication between the circulation and hypothalamic circuits [11]. However, their involvement in metformin-sensitive VMH Rap1 signaling has not yet been established.

Metformin remains a first-line therapy for T2DM, but its mechanisms extend beyond a single molecular target. Its metabolic actions involve changes in cellular energy status, mitochondrial function, and AMP-activated protein kinase (AMPK) signaling [12,13], together with AMPK-independent effects on hepatic glucose metabolism [14]. Metformin also acts beyond the liver, with reported effects involving the intestine, adipose tissue, immune system, gut microbiota, and CNS [15]. Within the brain, metformin may influence energy sensing, inflammatory responses, mitochondrial function, and neuronal activity [16]. Moreover, established hypothalamic and autonomic pathways provide a physiological route through which central metabolic sensing can influence peripheral glucose regulation [17]. These observations have increased interest in the brain as an additional site contributing to metformin action [18].

Within this emerging framework, Ras-related protein 1 (Rap1) represents a particularly relevant signaling candidate. Rap1 is a small GTPase that cycles between inactive GDP-bound and active GTP-bound states and functions as a context-dependent regulator of intracellular signaling [19]. In addition to its established roles in cell adhesion and signal transduction, Rap1 is involved in neuronal signaling and metabolic regulation, providing a mechanistic basis for investigating its contribution to central metformin action [20]. Experimental studies suggest that hypothalamic Rap1 affects leptin sensitivity, energy balance, and glucose homeostasis [21]. Moreover, the cAMP–Epac–Rap1 pathway may also impair hypothalamic hormone responsiveness during the excess of nutrients [22]. These findings provide a biological basis to investigate whether metformin can modulate Rap1-dependent neuronal pathways.

Recent review by Ahmad and Akhtar (2026) described the CNS actions of metformin, including the emerging VMH–SF1–Rap1 mechanism [23]. The present review therefore does not aim merely to reiterate this pathway. Instead, it critically examines the metformin–Rap1 axis from a mechanistic-to-translational perspective by distinguishing experimentally established Rap1-dependent effects from established non-Rap1 neural pathways and proposed brain-to-organ connections. Particular emphasis is placed on dose and CNS exposure, the emerging but incompletely defined Rap1–AMPK relationship, and the distinction between peripheral glucose disposal and hepatic glucose regulation. The review further integrates recent human evidence, including CSF pharmacokinetics, functional CNS observations, RAP1A pharmacogenetics, and the parallel GDF15–GFRAL pathway, to develop a translational evidence framework for evaluating human Rap1 target engagement. Finally, it considers pathway- and circuit-selective modulation and defines the experimental steps required to determine whether brain Rap1 can progress from a preclinical mechanism to a clinically relevant biomarker or therapeutic target in T2DM.

## 2. Literature Search Strategy

This narrative review was created by adopting a focused search of PubMed/MEDLINE, Scopus, and Google Scholar between January 2000 and September 2026. “Metformin,” “Rap1,” “hypothalamus,” “ventromedial hypothalamus,” “AMPK,” “glucose homeostasis,” “brain–liver axis,” and “type 2 diabetes,” were used as search terms. Related original research articles, clinical studies, and reviews were included. The reference lists of important publications were scrutinized to identify additional studies. Recent studies were given priority, whereas basic studies were retained to provide mechanistic context. The literature was synthesized narratively, with emphasis on separating established experimental findings from emerging hypotheses. No formal meta-analysis or quantitative assessment of study quality was performed. During the preparation of this manuscript, the authors used ChatGPT (version GPT-4o) for the purposes of improving the spelling, grammar, graphical abstract, figures and overall readability of the manuscript. The authors have reviewed and edited the output and take full responsibility for the content of this publication.

## 3. Rap1 Signaling Pathway

### 3.1. Structure and Regulation of Rap1 Signaling

Rap1 is a molecular switch that links extracellular signals to intracellular responses [18]. It exists as two closely related isoforms, Rap1A and Rap1B, which cycle between inactive GDP-bound and active GTP-bound states [24]. Although highly homologous and sharing many downstream effectors, the two isoforms may differ in their expression, subcellular distribution, and functions depending on cell type and signaling context. However, their isoform-specific roles in metabolically relevant hypothalamic neurons remain poorly understood. Rap1 signaling is also spatially regulated, as membrane-associated and intracellular Rap1 pools can engage distinct signaling partners. Such isoform- and compartment-specific regulation may be important for understanding brain Rap1 signaling and developing selective therapeutic strategies.

Rap1 regulates several cellular processes, including cell adhesion, cytoskeletal organization, vesicular trafficking, neuronal signaling, and metabolic responses [25]. Its activity is controlled by guanine nucleotide exchange factors (GEFs), which promote GTP loading, and GTPase-activating proteins (GAPs), which accelerate GTP hydrolysis. Rap1 GEFs include C3G, PDZ-GEFs, CalDAG-GEFs, and exchange proteins directly activated by cAMP (Epac), whereas Rap1GAP and signal-induced proliferation-associated proteins contribute to its inactivation [25,26]. The cAMP–Epac–Rap1 axis is particularly relevant to neuronal and metabolic signaling. Epac1 and Epac2 function as cAMP sensors that activate Rap proteins independently of protein kinase A (PKA). Activated Rap1 can engage B-Raf–MEK–ERK signaling and interact with PI3K/Akt, integrins, and Ca^2+^-dependent pathways [27,28]. Thus, Rap1 functions as a spatially and context-dependent signaling hub rather than a simple linear pathway, an important consideration for selective therapeutic targeting.

### 3.2. Rap1 Signaling in the Brain

Rap1 is widely distributed in various parts of brain, including the cortex, hippocampus, hypothalamus, striatum, amygdala, and cerebellum. Human brain studies have demonstrated region-specific expression of Rap1 and Epac [29]. The broad distribution of Rap1 reflects its involvement in neuronal excitability, synaptic plasticity, hormonal responses, and metabolic regulation. The metabolic significance of Rap1 becomes more relevant during nutrient excess. Kaneko et al. (2016) showed that high-fat feeding increases neuronal Rap1 activity and that neuronal Rap1 deficiency can attenuate diet-induced obesity, peripheral insulin resistance, and leptin resistance [21]. Pharmacological inhibition of brain Rap1 has ability to improve metabolic and leptin responses. The cAMP–Epac–Rap1 pathway also impacts hypothalamic hormone responsiveness. Activation of cAMP–Epac signaling can impair hypothalamic leptin signaling and reduce leptin responsiveness in POMC neurons [22]. In parallel, gut-derived glucose-dependent insulinotropic polypeptide (GIP) can stimulate central Rap1 and decrease hypothalamic leptin sensitivity [22,24]. These observations keep Rap1 as an interface between peripheral nutritional signals and central metabolic responsiveness.

**Figure 1:**
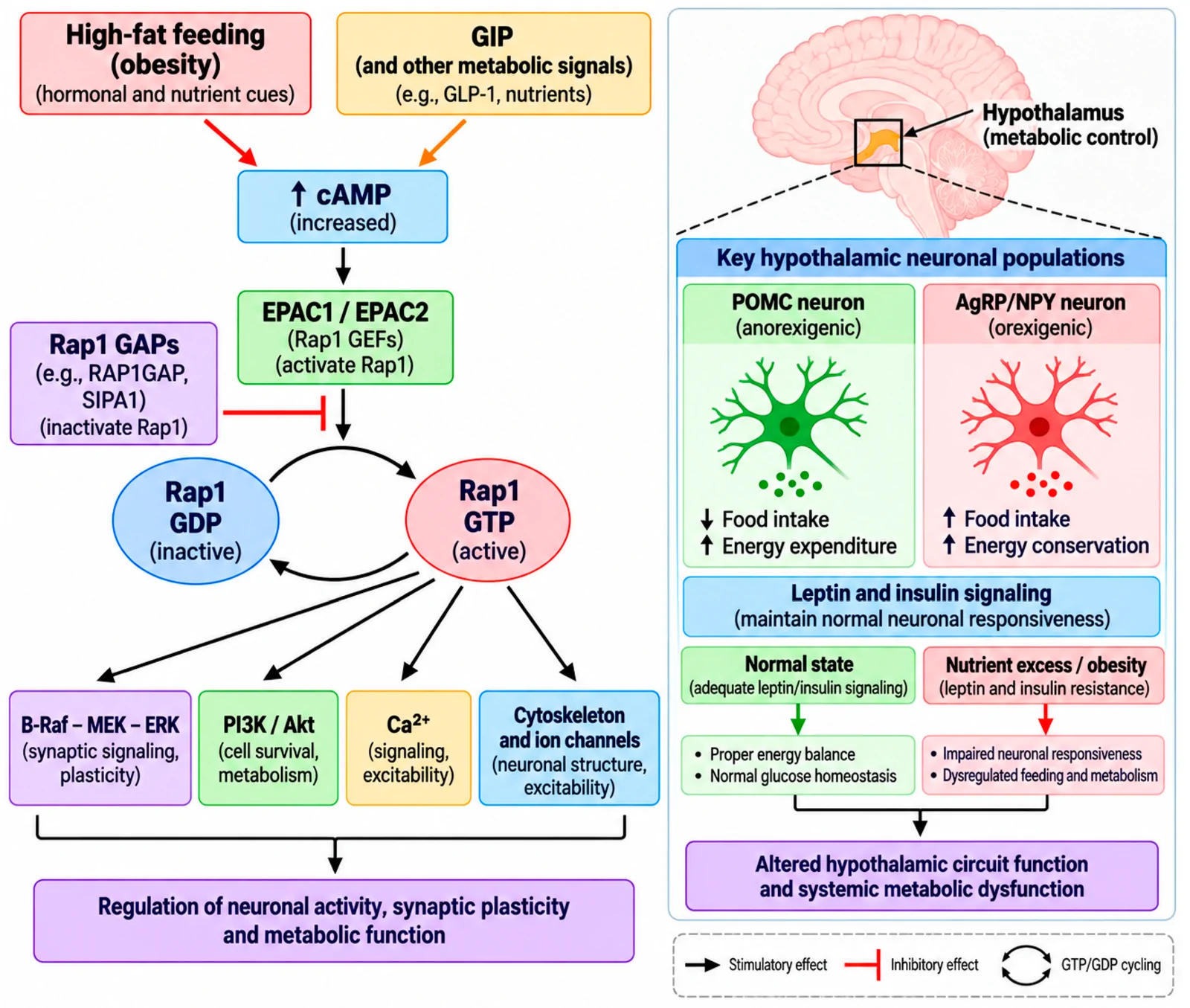
Molecular regulation of Rap1 signaling in the brain. Rap1 cycles between GDP-bound inactive and GTP-bound active states under GEFs and GAPs control. Nutrient-related signals may activate the cAMP–EPAC–Rap1 pathway, which interacts with ERK, PI3K/Akt, calcium, and cytoskeletal signaling to regulate neuronal activity and metabolism. An excessive Rap1 signaling in the hypothalamus during overnutrition may impair leptin responsiveness, contributing to metabolic dysfunction.

Morozov et al. (2003) demonstrated that disruption of forebrain Rap1 signaling impaired cAMP-dependent hippocampal long-term potentiation and contextual discrimination [25]. Mechanistically, Rap1 can link cAMP signaling to the extracellular signal-regulated kinase 1/2 (ERK1/2) pathway and influence phosphorylation of the A-type potassium channel Kv4.2. Rap1 can also integrate Ca^2+^-dependent signaling with B-Raf–ERK activation in neurons [26]. Together, these findings indicate that Rap1–ERK signaling can influence neuronal excitability and synaptic plasticity. However, these broader neuronal functions should not be interpreted as evidence that ERK mediates the glucose-lowering effects of metformin downstream of VMH Rap1, as this relationship has not been directly established. Collectively, the available evidence supports Rap1 as an activity- and nutrient-sensitive neuronal signaling node whose metabolic effects are strongly region- and cell-dependent. This provides a basis for investigating whether metformin engages specific hypothalamic Rap1 circuits to regulate glucose homeostasis. The principal regulatory mechanisms and metabolic functions of brain Rap1 are summarized in Figure 1.

## 4. Metformin and the Central Nervous System

### 4.1. Brain Penetration and CNS Pharmacokinetics

It is important for metformin to reach pharmacologically appropriate concentrations in the CNS. Because of its hydrophilic nature and positive charge, metformin does not readily diffuse through lipid membranes, and its distribution depends markedly on transporter-mediated processes. Nevertheless, systemic administration can result in detectable metformin concentrations in brain tissue and cerebrospinal fluid (CSF). Experimental studies provide useful information on CNS exposure. For example, in mice receiving intraperitoneal metformin at 200 mg/kg, CSF concentrations reached approximately 29 μM within 30 min and subsequently declined [27]. Following intravenous administration, LC-MS/MS analysis measured metformin in mouse brain within 5 min, with a blood-to-brain influx constant of 1.87 μL/g/min [28]. These results indicate that metformin can enter the CNS, but CSF concentrations should not be presumed to signify total brain-tissue or intracellular exposure [29]. Drug distribution may differ with dose, route, treatment duration, blood–brain barrier integrity, and disease state [30].

A comparison of exposure throughout experimental settings is crucial for inferring the proposed Rap1 mechanism. Therapeutic concentration in plasma is generally within the low-micromolar range, whereas human CSF values have demonstrated markedly lower concentrations. In contrast, animal experiments by using relatively high systemic metformin doses may produce CSF concentrations in the tens of micromolar range, and brain-tissue measurements may differ from CSF values. Recent Rap1-focused work has examined responses to micromolar metformin, whereas earlier studies using millimolar concentrations may not reflect clinically relevant exposure. These differences suggest that a central Rap1 mechanism may be pharmacologically plausible, particularly if the relevant neuronal response occurs at lower concentrations. However, it remains uncertain whether therapeutic dosing achieves sufficient concentrations metformin attains adequate concentrations in human VMH neurons. Plasma and CSF values do not establish regional brain exposure, intracellular accumulation, or direct Rap1 target engagement. Studies combining clinically relevant dosing, regional brain pharmacokinetics, and concentration-dependent measurements of Rap1 activity are therefore needed to know whether the proposed pathway contributes meaningfully to metformin’s glucose-lowering effects.

### 4.2. Context-Dependent Neuronal AMPK Signaling

AMP-activated protein kinase (AMPK) is a major mediator of cellular energy sensing and an established target of metformin [31]. In neural cells, metformin can modify mitochondrial energetics and AMPK phosphorylation, thereby influencing mitochondrial function, protein synthesis, autophagy, and survival. Metformin protected PC12 cells and primary hippocampal neurons against H_2_O_2_-induced oxidative injury through an AMPK-dependent mechanism [32]. Similarly, metformin improved mitochondrial function in AGE-exposed human neural stem cells, restoring ATP production, mitochondrial mass, membrane potential, and expression of mitochondrial regulators [33].

AMPK can also influence mTORC1 and autophagy. In diabetic db/db mice and high-glucose-exposed neuronal cells, metformin improved autophagic clearance and reduced tau hyperphosphorylation [34]. Similar AMPK/mTORC1-associated autophagic effects have been reported in dopaminergic neurons under mitochondrial stress [35]. However, neuronal AMPK responses are not uniform. In primary hypothalamic neurons under low-glucose conditions, metformin inhibited AMPK phosphorylation and prevented the increase in neuropeptide Y expression [36]. Thus, the conventional model of uniform metformin-induced AMPK activation cannot be directly transferred from peripheral tissues to all neuronal populations. This context dependence may arise from differences among neuronal populations and brain regions. Neurons differ in their metabolic demands, cellular energy status, and expression of metformin transporters and AMPK-regulatory proteins. These factors may influence intracellular metformin exposure and changes in the AMP/ATP balance. In addition, AMPK can be regulated through lysosomal mechanisms and upstream kinases, which may vary according to neuronal type and physiological state. The dose and duration of metformin exposure may further influence these responses. Therefore, metformin-induced AMPK activation should be considered cell- and context-dependent rather than a uniform response throughout the CNS.

### 4.3. Neuroinflammation, Oxidative Stress, and Mitochondrial Protection

Metformin also acts through AMPK-independent mechanisms involving mitochondrial function, redox signaling, inflammatory pathways, and cell survival. In primary cortical neurons, it delayed mitochondrial permeability-transition-pore opening and limited mitochondria-associated apoptotic signaling [37]. In spinal neurons, metformin reduced H_2_O_2_-induced oxidative and inflammatory responses through modulation of P2X7 receptor signaling [38]. Microglia represent another relevant CNS target. In LPS-activated primary microglia, metformin reduced inflammatory and oxidative mediators through a mixture of AMPK-dependent and AMPK-independent mechanisms [39]. In a cuprizone model, metformin reduced microgliosis and astrogliosis while suppressing NF-κB-associated inflammatory signaling [40]. Metformin can furthermore decrease ROS, conserve mitochondrial membrane potential, and augment antioxidant defenses [16,41]. Thus, metformin reaches the CNS and alters neuronal and glial signaling through various mechanisms. This wider pharmacological background is essential, but these effects should not be interpreted as evidence of Rap1 dependence.

## 5. Emerging Role of Brain Rap1 in Metformin’s Antidiabetic Action

Preclinical evidence has begun to find a role for brain Rap1 as a potential mediator of the glucose-lowering action of metformin. Earlier studies established Rap1 as a nutrient-responsive regulator of hypothalamic metabolism and identified the VMH as an important site for Rap1-dependent glycemic regulation [21,42]. More recently, Lin et al. (2025) provided experimental evidence that suppression of brain Rap1 contributes to the glucose-lowering response to low-dose metformin [43]. While these findings offer important mechanistic support, the evidence remains largely preclinical and is derived from a limited number of experimental studies.

### 5.1. Metformin Targets the VMH Rap1 Pathway

Kaneko et al. (2021) demonstrated that greater Rap1 activity in the medial hypothalamus raised glucose in high-fat-diet-fed mice [42]. In contrast, selective deletion of Rap1A and Rap1B from steroidogenic factor-1-positive (SF-1) neurons in the VMH reduced circulating glucose and insulin and improved glucose tolerance and peripheral insulin sensitivity. These findings established VMH Rap1 as a regulator of glucose homeostasis, which can be independent of body-weight control.

Intracerebroventricular administration of metformin decreased active Rap1 in the hypothalamus and lowered blood glucose in diet-induced obese mice. Mice with forebrain-specific deletion of Rap1A/Rap1B, exhibited markedly reduced glucose-lowering response at lower metformin doses while retaining responsiveness to other antidiabetic drugs, supporting a selective requirement for Rap1 in metformin action [43]. Gain-of-function experiments provided complementary evidence as constitutive activation of neuronal Rap1 increased glycemia and impaired glucose tolerance, while maintaining Rap1 in an active state markedly reduced the glucose-lowering effect of metformin. Similarly, Rap1 deletion in SF-1 neurons reproduced much of the glucose-lowering phenotype associated with metformin, whereas constitutive VMH Rap1 activation attenuated metformin-induced improvements in glycemia. Together, these experiments provide preclinical evidence that VMH Rap1 suppression contributes importantly to the glucose-lowering action of low-dose metformin. However, confirmation in additional experimental models and ultimately in humans is required.

### 5.2. Rap1-Dependent Modulation of Neuronal Excitability

**Figure 2:**
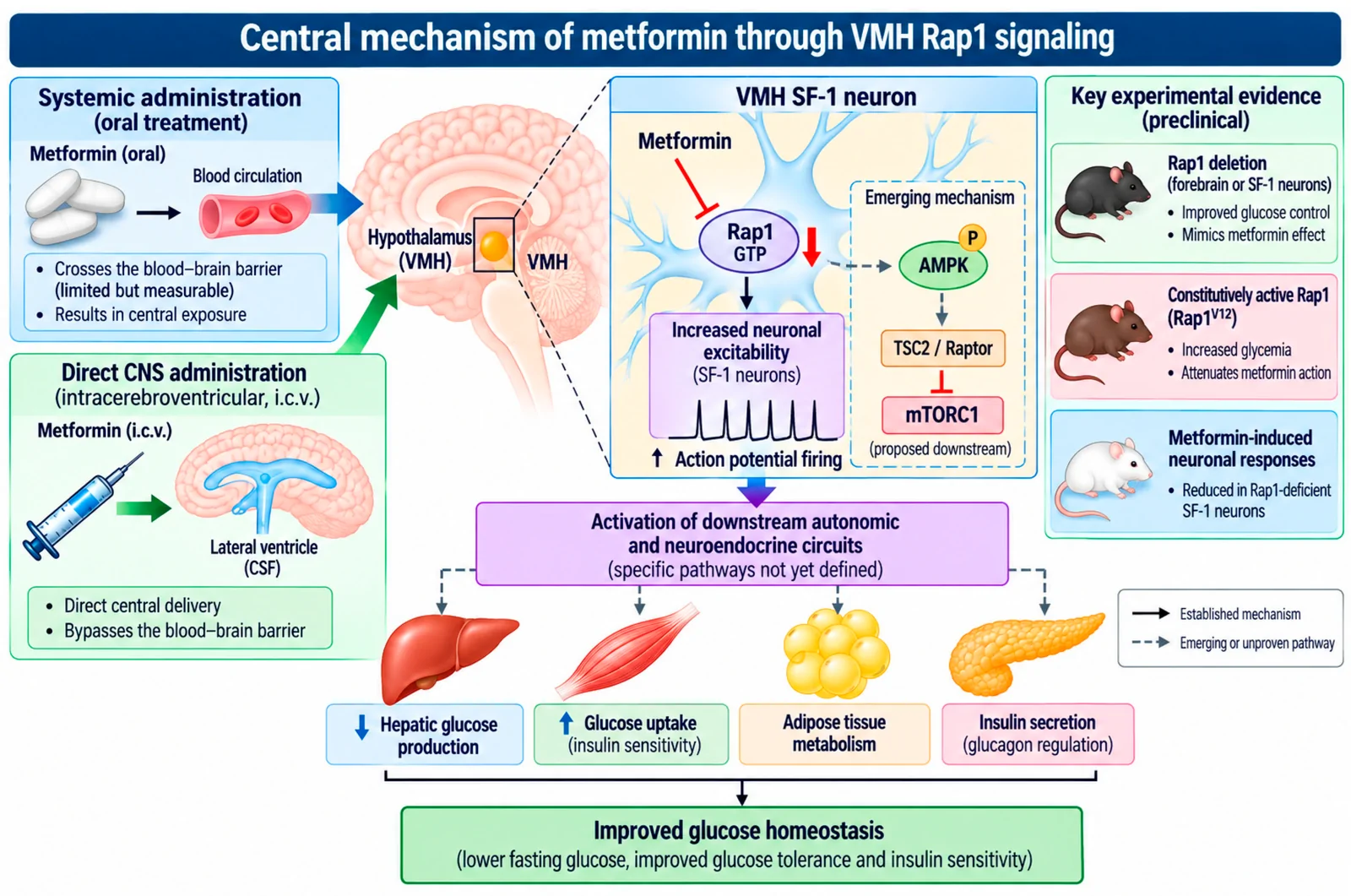
Emerging central mechanism of metformin through VMH Rap1 signaling. Systemically administered metformin may reach the CNS after oral treatment, whereas intracerebroventricular (i.c.v.) administration provides direct central delivery and bypasses the blood–brain barrier. Preclinical studies suggest that metformin inhibits active Rap1 in VMH SF-1 neurons and alters neuronal excitability, contributing to improved glucose regulation. Genetic deletion and constitutive activation of Rap1 provide complementary evidence for its involvement in the response to low-dose metformin. Rap1–AMPK represents an emerging functional relationship, whereas mTORC1 and the specific autonomic and neuroendocrine pathways linking VMH Rap1 to peripheral metabolic organs remain proposed mechanisms requiring further validation. Solid arrows indicate experimentally supported relationships, whereas dashed arrows indicate emerging or proposed pathways.

There is a possible cellular bridge between Rap1 inhibition and glucose control. Metformin administration preferentially elevated c-Fos expression in the VMH. Whole-cell recordings from identified SF-1 neurons further demonstrated that micromolar metformin depolarized responsive neurons and increased action-potential firing. Importantly, Rap1 deletion impaired metformin-induced electrophysiological responses. These findings suggest that Rap1 may function as a molecular link between metformin exposure and altered activity of VMH neurons. The intracellular mechanisms linking Rap1 inhibition to membrane depolarization and subsequent signaling to peripheral tissues remain unclear. The mechanism of metformin action through VMH Rap1 signaling and the supporting experimental evidence are summarized in Figure 2.

### 5.3. Emerging Rap1–AMPK Signaling

Recent preliminary evidence suggests a possible functional relationship between hypothalamic Rap1 and AMPK. However, these findings are currently available only as a conference abstract and should be interpreted cautiously until independently validated in a full peer-reviewed study. In contrast, peer-reviewed evidence supports the involvement of VMH Rap1 in the glucose-lowering action of low-dose metformin, but does not establish AMPK as a downstream mediator of Rap1. Lu et al. (2026) suggested a possible relationship between Rap1 and AMPK in the VMH [44]. BioID proximity proteomics identified AMPK-associated proteins among potential Rap1-interacting components, while metformin increased AMPK phosphorylation approximately 2.26-fold in the VMH. Neuronal AMPK deletion abolished the glucose-lowering response to metformin, and Rap1 deletion was associated with elevated AMPK phosphorylation. Together, these observations suggest that Rap1 may influence AMPK activity and that neuronal AMPK may contribute to the central metabolic response to metformin. However, these findings are currently available as a conference abstract, and the direction, molecular basis, and cell-specific nature of the relationship remain to be established. In particular, proximity proteomics does not demonstrate direct Rap1–AMPK binding, and the available evidence does not establish a complete signaling sequence.

Low-dose metformin-induced activation of lysosomal AMPK is mediated through PEN2 and ATP6AP1 [45]. Whether Rap1 influences this machinery in VMH SF-1 neurons, or acts through a distinct intermediate pathway, remains unknown. Separately, AMPK is known to regulate mTORC1 through TSC2 and Raptor [46]. This established relationship provides a rationale for investigating mTORC1 as a potential downstream component, but there is currently no direct evidence that metformin-induced Rap1 inhibition regulates hypothalamic mTORC1. Accordingly, Rap1–AMPK should be regarded as an emerging functional relationship, whereas involvement of mTORC1 remains a separate mechanistic possibility requiring direct experimental validation.

### 5.4. Current Mechanistic Limitations

The proposed metformin–Rap1 mechanism is supported mainly by preclinical studies, particularly genetically modified mouse models. Although loss- and gain-of-function studies provide mechanistic support, independent replication remains limited and direct evidence in humans is lacking. The most strongly supported sequence is currently: low-dose metformin → inhibition of VMH Rap1 → altered SF-1 neuronal excitability → improved glucose homeostasis. The Rap1–AMPK relationship remains emerging, while specific downstream brain-to-organ circuits are not yet defined. These knowledge gaps and the experimental approaches needed to address them are discussed in Section 8.

## 6. Potential Systemic Implications: From Central Rap1 Signaling to Inter-Organ Metabolic Control

The physiological relevance of central Rap1 signaling depends on how changes in hypothalamic neuronal activity influence peripheral glucose metabolism. The hypothalamus communicates with metabolic organs through autonomic and neuroendocrine pathways [9,47]. However, the specific circuits recruited by metformin-sensitive VMH Rap1 neurons remain largely undefined. Therefore, established neural pathways should provide a framework for investigation rather than be considered components of a confirmed Rap1-dependent pathway.

### 6.1. Established Rap1-Dependent Metabolic Effects

Experimental evidence suggests that VMH Rap1 contributes to systemic glucose regulation. Deletion of Rap1 in VMH SF-1 neurons improves glucose tolerance and insulin sensitivity [42]. Hyperinsulinemic–euglycemic clamp studies further demonstrate increased peripheral glucose disposal, with enhanced glucose uptake in skeletal muscle and adipose tissue. Thus, peripheral glucose utilization currently represents the strongest direct systemic metabolic effect associated with VMH Rap1 inhibition. Neuronal Rap1 has also been linked to energy balance and diet-induced obesity [21]. However, the metabolic functions of Rap1 appear to depend on neuronal population and anatomical site. In the VMH, Rap1 deletion increases glucose disposal in adipose tissue, but whether metformin-sensitive VMH Rap1 directly regulates adipose lipolysis, lipogenesis, or fatty-acid handling remains unclear [42]. Low-dose metformin also suppresses hypothalamic Rap1 activity and alters SF-1 neuronal excitability. These effects may contribute to its glucose-lowering action. However, whether metformin-induced Rap1 inhibition reproduces the tissue-specific metabolic effects observed after genetic Rap1 deletion remains unclear. Establishing this connection should therefore be a major experimental priority.

### 6.2. Established Non-Rap1 Neural Pathways

Several established neural pathways regulate peripheral glucose metabolism independently of Rap1. Hypothalamic insulin signaling can suppress endogenous glucose production [48], with KATP channels and autonomic signaling contributing to this response [49]. Hypothalamic nutrient sensing can similarly regulate hepatic glucose flux through KATP-dependent and autonomic mechanisms [50]. Metformin can also influence hepatic glucose production through an intestinal neural pathway. Duca et al. demonstrated that duodenal metformin activates mucosal AMPK and engages GLP-1 receptor–PKA signaling, intestinal afferent pathways, NTS NMDA receptors, and hepatic vagal signaling [51]. This gut–brain–liver pathway is experimentally established but should remain distinct from the emerging VMH Rap1 mechanism. Importantly, VMH Rap1-dependent glucose regulation can occur independently of major changes in body weight. In experimental models, increasing medial hypothalamic Rap1 activity worsened hyperglycemia without increasing body weight, whereas reducing Rap1 activity improved glycemic control without preventing diet-induced weight gain [42]. These findings suggest that the glycemic effects of VMH Rap1 involve direct regulation of glucose metabolism and insulin sensitivity rather than simply resulting from reduced food intake or weight loss.

### 6.3. Priority Pathways for Experimental Validation

Neural and autonomic pathways can affect skeletal-muscle glucose utilization, adipose metabolism, pancreatic hormone secretion, and hepatic glucose flux [52,53]. Metformin may also exert both direct peripheral and CNS-mediated metabolic effects [54]. However, the specific circuits linking metformin-responsive VMH Rap1 neurons to peripheral organs remain undefined. The strongest direct metabolic evidence is the increased peripheral glucose disposal observed following VMH Rap1 deletion [42]. Therefore, the first priority should be to determine whether metformin-sensitive VMH Rap1 neurons regulate glucose uptake and insulin action in skeletal muscle and adipose tissue through similar mechanisms.

**Figure 3:**
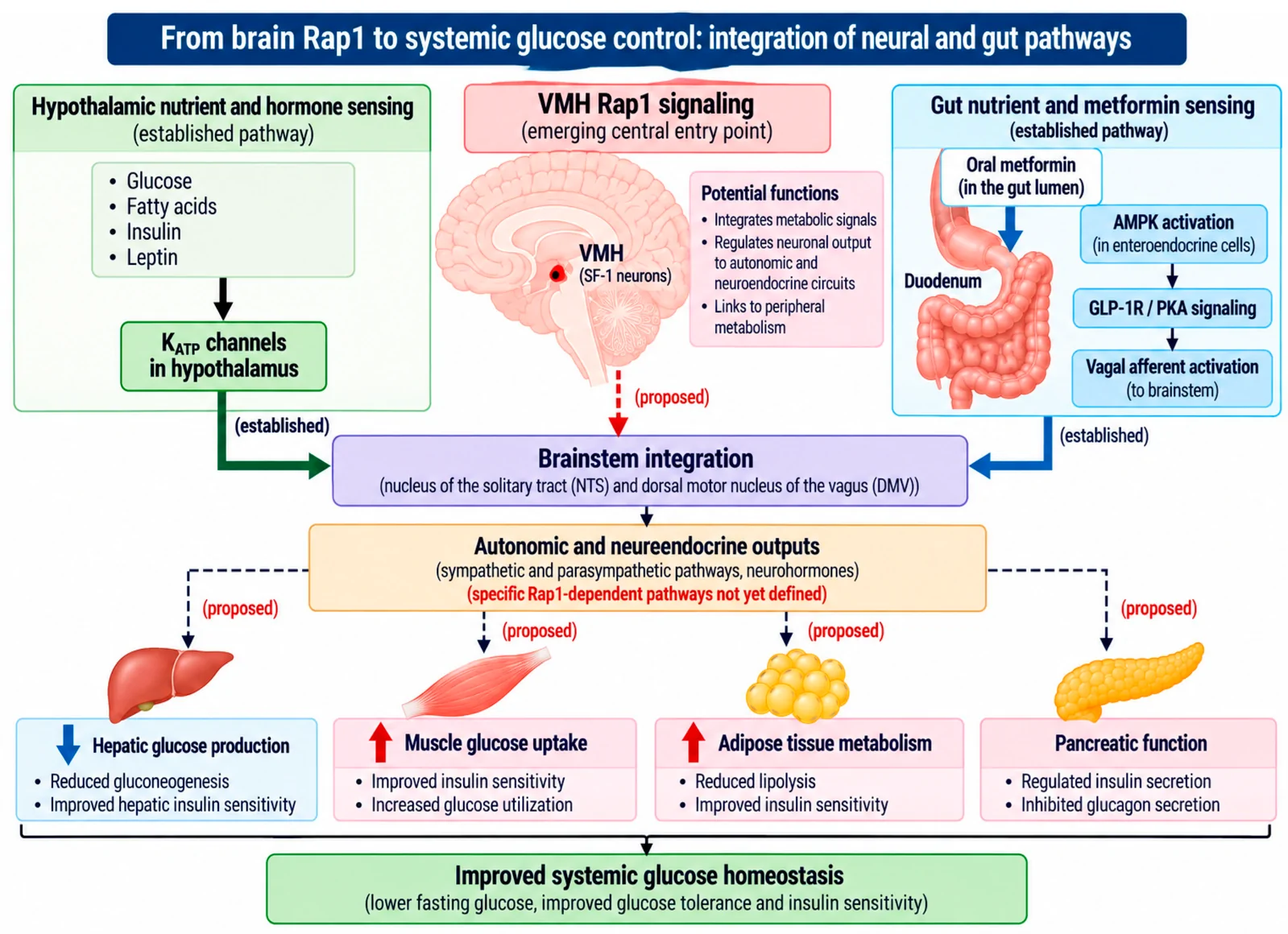
Neural and gut pathways integrate through brain to glucose control. This figure elucidates established hypothalamic and intestinal pathways through which nutrient and metformin sensing may affect glucose production and its peripheral metabolism. KATP-dependent signaling (in hypothalamus) and AMPK–GLP-1R/PKA signaling (in duodenum) can employ brainstem and autonomic circuits to normalize glucose homeostasis. VMH Rap1 represents an emerging potential entry point that may interact with these broader neuro-metabolic networks. The downstream pathways that connect metformin-sensitive Rap1 neurons to peripheral organs remain to be partly defined. This diagram is represented as a simplified conceptual framework, not a fully established Rap1-dependent circuit.

A second priority is to determine whether hypothalamic KATP channels, brainstem relays, and autonomic pathways contribute to Rap1-mediated glucose regulation. These pathways are established components of central metabolic control [51,55], but their involvement downstream of VMH Rap1 has not been demonstrated. Cell-specific Rap1 manipulation combined with neural tracing, targeted circuit interventions, and tissue-specific glucose-flux measurements could directly test these connections. Effects on hepatic glucose production, pancreatic hormone secretion, and other neuroendocrine pathways should remain secondary questions until stronger evidence links them to VMH Rap1 [52,53,55]. Thus, established hypothalamic and intestinal pathways provide a physiological framework, but not proof, for Rap1-dependent inter-organ communication. These established and proposed pathways are summarized in Figure 3.

## 7. Clinical Relevance and Translational Potential

Clinical translation of the proposed metformin–Rap1 mechanism requires evidence that this central pathway is engaged in humans and contributes meaningfully to glycemic regulation. Direct evidence that metformin inhibits Rap1 activity in the human hypothalamus is currently lacking. Nevertheless, human studies demonstrate that metformin reaches the CNS and can influence molecular and functional brain outcomes, providing biological plausibility for a central component of its action.

### 7.1. Human Evidence for Central Metformin Activity

In a randomized crossover study of nondiabetic adults with mild cognitive impairment or mild Alzheimer’s disease, orally administered metformin was detected in the CSF. Metformin showed possible improvement in executive function, but no significant overall changes in regional cerebral blood flow were observed [56]. Subsequent proteomic studies recognized changes in 50 plasma proteins and 26 CSF proteins, with about seven proteins varying in both compartments [29]. These results give evidence of CNS exposure and molecular responses, but do not prove Rap1 engagement. Functional neuroimaging provides complementary proof. Schizophrenia patients receiving antipsychotics and adjunctive metformin for 24 weeks improved several cognitive domains and improved functional connectivity between dorsolateral prefrontal cortex subregions and anterior/middle cingulate areas. These connectivity changes are correlated with the improvements in selected cognitive measures [57]. Although this population is different from T2DM, the findings indicate that systemic metformin can affect human brain-network performance. Similarly, Luchsinger et. al. demonstrated an improved total recall after 12 months of metformin in overweight or obese adults with amnestic mild cognitive impairment, although several secondary outcomes, including FDG-PET measures, were unchanged [58].

These findings can be explained within a translational evidence hierarchy. Primarily, the analysis of metformin in CSF confirms CNS exposure, but does not define regional or intracellular concentrations. Secondly, proteomic, neuroimaging, and cognitive alterations give evidence of functional CNS responses, although they are not particular to Rap1. Third, genetic associations including RAP1A may offer pathway-related support, but they cannot impartially establish a central mechanism. Furthermore, a direct target engagement would need evidence that clinically relevant metformin exposure changes Rap1 activity in human hypothalamic neurons. Lastly, clinical benefit would require potential evidence that this engagement contributes to meaningful glycemic improvement outside established peripheral mechanisms. At present, the available human studies primarily support the first two levels, while pharmacogenetic findings provide preliminary support at the third. Direct human hypothalamic Rap1 engagement and its contribution to clinical glucose lowering remain to be established. This distinction highlights the need for integrated pharmacokinetic, molecular, neuroimaging, and metabolic studies before the proposed pathway can be considered clinically validated.

### 7.2. Neurological Outcomes: Supportive but Heterogeneous Evidence

Observational evidence is similarly mixed. In the Sydney Memory and Ageing Study, older adults with diabetes receiving metformin showed slower decline in global cognition and executive function, with a lower incidence of dementia than diabetic participants not receiving metformin [59]. However, meta-analytic findings have not consistently supported neuroprotection. A meta-analysis of 10 observational studies involving 229,110 participants found no significant overall reduction in Alzheimer’s disease incidence, while an increased association was reported in Asian participants [60]. Other studies have also reported neutral or adverse cognitive associations. Such heterogeneity emphasizes that neurological outcomes should not be used as surrogate evidence of brain Rap1 engagement. Age, metabolic status, diabetes duration, renal function, vitamin B12 status, concomitant medications, and genetic differences may all modify CNS responses to metformin.

### 7.3. RAP1A Pharmacogenetics

The most direct human evidence linking Rap1 to metformin responsiveness currently comes from pharmacogenetics. Lehrer and Rheinstein (2026) examined the intronic RAP1A variant rs7525578 in UK Biobank participants with T2DM. Among 7002 men receiving metformin, CT carriers had modestly higher HbA1c than CC carriers [61]. The association was not observed among pioglitazone or gliclazide users, suggesting possible drug specificity. The effect was small, was not observed in women, and did not directly assess brain Rap1 activity. It should, therefore, be considered initial pharmacogenetic support rather than clinical validation of the hypothalamic mechanism. Nevertheless, it raises the possibility that Rap1-pathway variation contributes to interindividual differences in metformin response.

### 7.4. Parallel CNS Pathways and Heterogeneous Metformin Responses

Rap1 is unlikely to represent the only neural pathway contributing to metformin action. The GDF15–GFRAL system provides a clear example. Coll et al. (2020) demonstrated that metformin increases circulating GDF15 in humans and that GDF15 signaling through the brainstem-restricted receptor GFRAL is required for much of metformin’s effect on food intake and body weight in mice [62]. These findings suggest a possible functional partitioning in which hindbrain GDF15–GFRAL signaling contributes strongly to appetite and body-weight responses, whereas hypothalamic Rap1 may be particularly relevant to glycemic regulation. This distinction remains provisional, and the relative contributions and interactions of these pathways in humans require direct investigation.

### 7.5. Pharmacological Tractability and Safety

Direct systemic inhibition of Rap1 is unlikely to be optimal because Rap1 regulates integrin signaling, platelet activation, immune-cell migration, vascular function, and neuronal plasticity [63]. Therapeutic development may therefore require targeting selected upstream regulators or Rap1–effector interactions. EPAC proteins provide proof that the pathway is pharmacologically tractable. Biochemical, structure–activity, and NMR analyses demonstrated that ESI-09 inhibits EPAC1 and EPAC2 at concentrations below those producing nonspecific protein destabilization [64]. Systemic ESI-09 has also produced physiological effects through inhibition of EPAC1–Rap1 signaling in inflammatory pain models [65].

**Figure 4:**
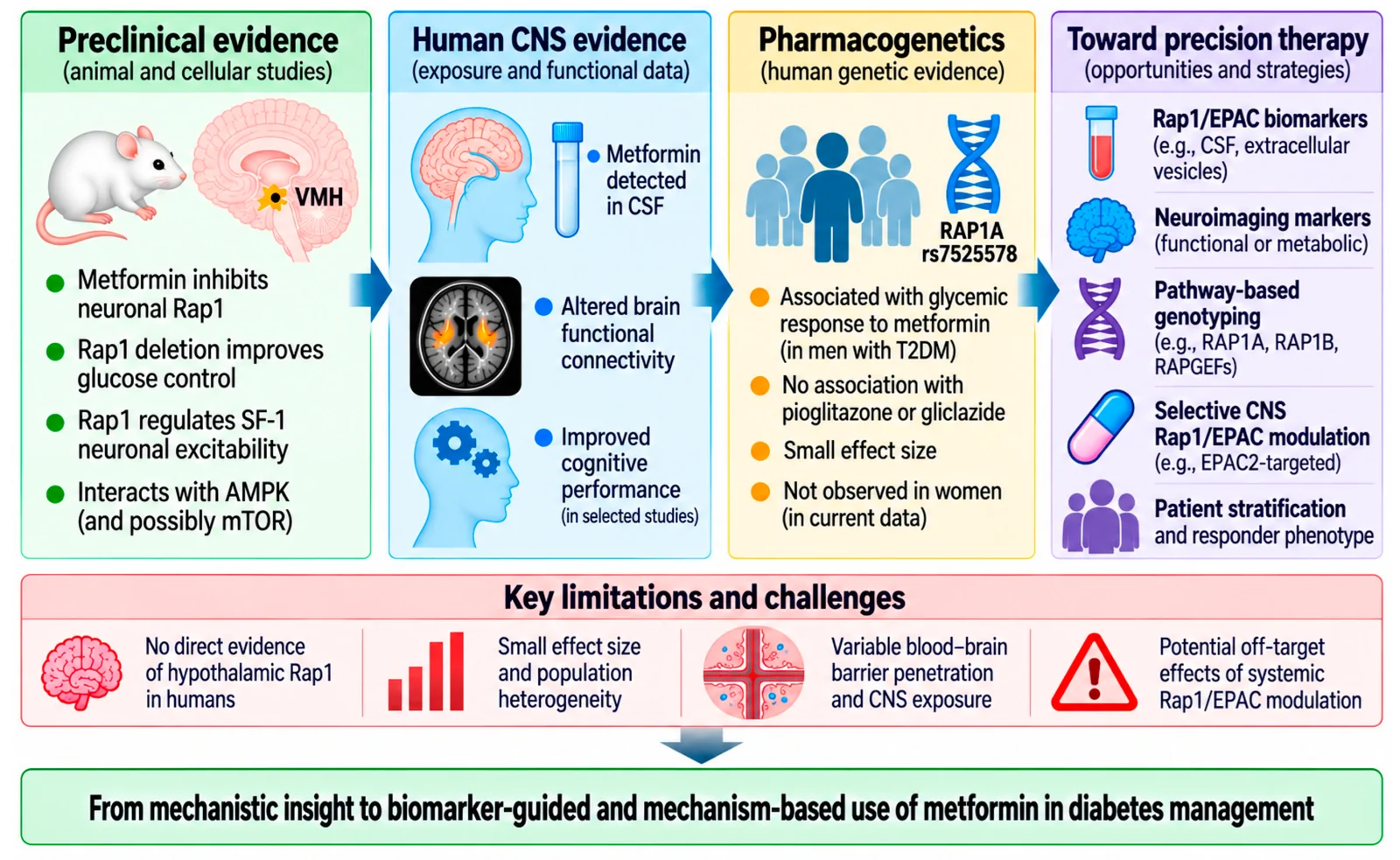
Clinical translation of the metformin–Rap1 axis extending from bench to bedside. The above figure demonstrates the progression from preclinical evidence of Rap1-dependent metformin action to human CNS observations, emerging RAP1A pharmacogenetic findings, and potential precision-medicine approaches. Human studies suggest that metformin can reach the CNS and affect functional brain outcomes, however, the direct evidence of hypothalamic Rap1 inhibition in humans is not yet available. Pathway-based biomarkers, genetic profiling, and selective CNS modulation may predict future opportunities to identify treatment-responsive individuals. The proposed approaches remain exploratory and require additional validation of efficacy and safety.

These findings support the feasibility of pharmacologically modulating the EPAC–Rap1 pathway, although they do not establish antidiabetic efficacy. EPAC2-directed approaches may be particularly relevant to neuronal Rap1 signaling, but systemic inhibition could also affect pancreatic β-cell signaling and neurotransmission. Similarly, maximizing BBB penetration without regional or pathway selectivity could produce unwanted neuronal, vascular, immune, or platelet effects. Overall, existing human studies demonstrate CNS exposure and biological effects of metformin, while RAP1A pharmacogenetics provides an initial pathway-specific signal. Direct evidence of human hypothalamic Rap1 engagement, however, remains the critical missing translational link. The available translational evidence, current limitations, and potential strategies for precision-based application of the metformin–Rap1 pathway are summarized in Figure 4.

## 8. Future Research Priorities

Future research should focus on three priorities: defining the molecular and circuit basis of metformin-sensitive Rap1 signaling, developing selective Rap1-directed strategies, and establishing human target engagement and clinical relevance.

### 8.1. Defining Molecular and Circuit Mechanisms

Future studies should determine how Rap1 inhibition influences AMPK activity and SF-1 neuronal excitability. Cell-specific experiments integrating Rap1-GTP measurements, AMPK signaling, and electrophysiology could help define the sequence of these events. The possible involvement of PEN2–ATP6AP1-mediated lysosomal AMPK signaling and mTORC1 should be tested directly rather than inferred from related pathways. Neural tracing and targeted circuit manipulation should then identify the downstream pathways used by metformin-responsive VMH neurons. These approaches should be combined with tissue-specific glucose-flux measurements, with peripheral glucose disposal in skeletal muscle and adipose tissue as an initial priority. The involvement of KATP-dependent, brainstem, and autonomic pathways should also be tested experimentally rather than assumed from established non-Rap1 mechanisms.

### 8.2. Developing Selective Rap1-Directed Strategies

Therapeutic studies should prioritize isoform-, pathway-, and cell-selective modulation of Rap1 rather than global inhibition. Characterizing Rap1A and Rap1B expression, subcellular localization, and signaling partners in metabolically relevant hypothalamic neurons may identify more selective targets. GEF–Rap1 and Rap1–effector interactions could provide additional points for intervention. CNS-targeted delivery may further improve selectivity. Candidate approaches should be evaluated for metabolic efficacy together with potential effects on neuronal, pancreatic, vascular, immune, and platelet functions.

### 8.3. Establishing Human Target Engagement and Precision Medicine

Human studies should determine whether clinically relevant metformin exposure alters hypothalamic Rap1 signaling and whether such changes are associated with glycemic response. Studies combining metformin pharmacokinetics, glucose-flux measurements, insulin sensitivity, neuroimaging, and validated CNS biomarkers may help address this question. Pharmacogenomic studies should extend beyond individual *RAP1A* variants to examine pathway-level genetic variation and its functional consequences. Patient-derived neuronal models may provide complementary mechanistic evidence. Ultimately, these approaches may help determine whether a reproducible Rap1-sensitive metformin-response phenotype exists and whether selective modulation of this pathway has clinical value.

## 9. Conclusions

Emerging preclinical evidence suggests that inhibition of hypothalamic Rap1 may contribute to the antidiabetic action of metformin. Studies in experimental models indicate that VMH Rap1 influences glycemic regulation independently of major changes in body weight and that Rap1 suppression contributes to the glucose-lowering response to low-dose metformin. Rap1-dependent changes in SF-1 neuronal excitability provide a possible cellular mechanism, while the relationship between Rap1 and AMPK remains emerging. These findings support a potential central mechanism that may complement, rather than replace, the established peripheral actions of metformin. However, the current evidence is based on a limited number of preclinical studies, and the precise Rap1-dependent brain-to-organ circuitry remains unresolved. Translation to humans is the major remaining challenge. Current clinical studies confirm CNS exposure and biological activity of metformin, while RAP1A pharmacogenetics provides preliminary pathway-specific evidence, but direct demonstration of hypothalamic Rap1 modulation in humans is lacking. Given the broad physiological functions of Rap1, future therapeutic development should emphasize pathway- and circuit-selective modulation rather than indiscriminate inhibition. Extending beyond previous reviews focused mainly on the preclinical VMH–Rap1 mechanism, this review integrates CNS pharmacokinetics, RAP1A pharmacogenetics, and human translational evidence to critically evaluate the pathway from mechanistic discovery toward potential clinical relevance. Establishing human target engagement, validated biomarkers, and genetically defined responder phenotypes will ultimately determine whether brain Rap1 becomes a clinically actionable target or a precision-medicine marker for metformin responsiveness.

## Data Availability

No new data were created or analyzed in this study. Data sharing is not applicable to this article.

## Abbreviations

AMPK, AMP-activated protein kinase; BBB, blood–brain barrier; CNS, central nervous system; CSF, cerebrospinal fluid; EPAC, exchange protein directly activated by cAMP; GDF15, growth differentiation factor 15; GFRAL, GDNF family receptor alpha-like; GIP, glucose-dependent insulinotropic polypeptide; KATP, ATP-sensitive potassium channel; mTORC1, mechanistic target of rapamycin complex 1; POMC, pro-opiomelanocortin; Rap1, Ras-related protein 1; SF-1, steroidogenic factor-1; T2DM, type 2 diabetes mellitus; VMH, ventromedial hypothalamus.
